# Supplementary material for: Integration of Transcriptional Signatures from Brain Tissue and Plasma Extracellular Vesicles of a Preclinical Tauopathy Mouse Model
Source: Int J Mol Sci. 2026 Jun 3;27(11):5050. doi: 10.3390/ijms27115050 (PMC13256833; doi:10.3390/ijms27115050)
Supplement: Supplementary file 1 [file ijms-27-05050-s001.zip › ijms-4336543-supplementary/Supplementary files/Supplemtal Figure S1 Legends.pdf]

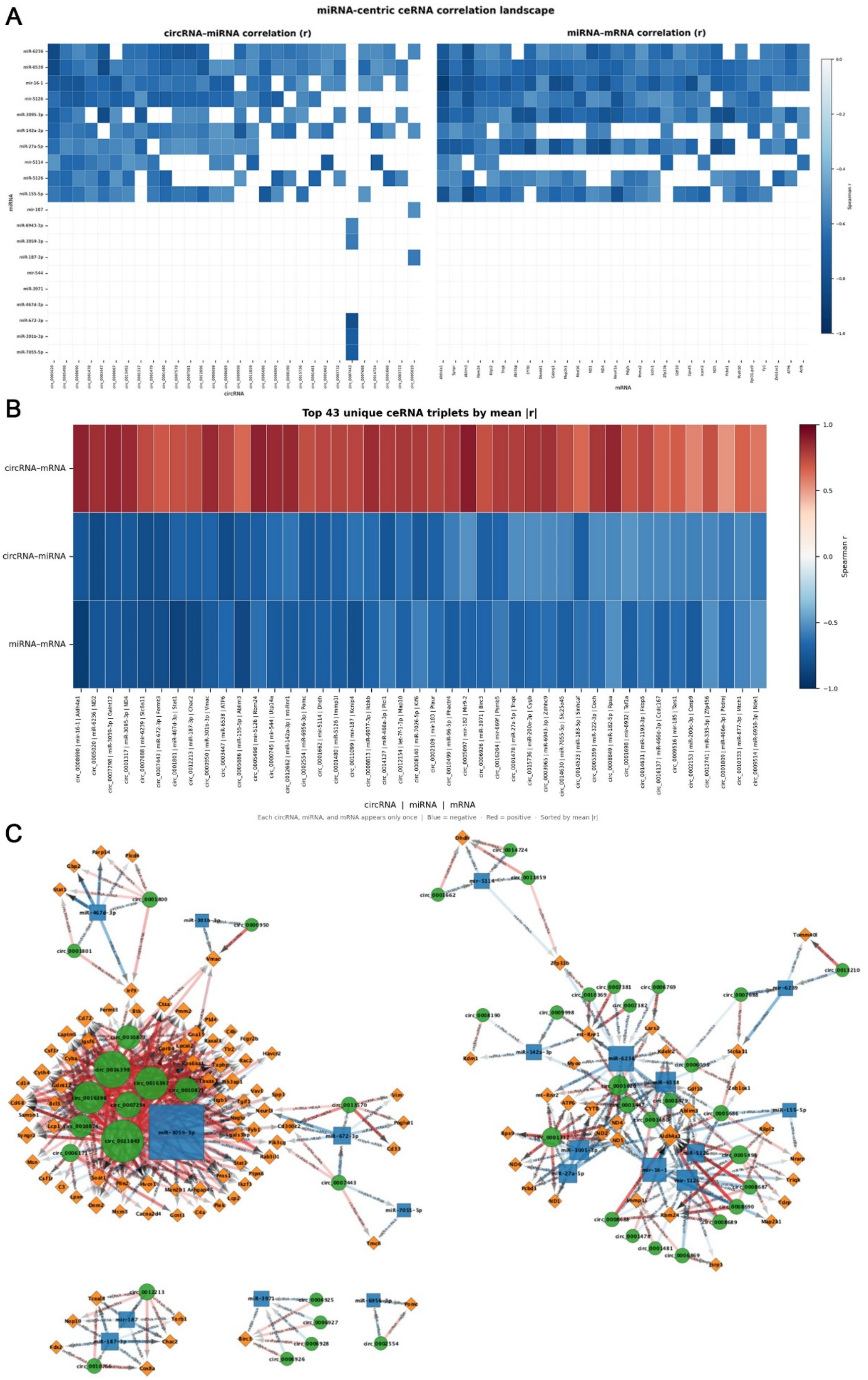
A
miRNA-centric ceRNA correlation landscape
circRNA–miRNA correlation (r)
miRNA–mRNA correlation (r)
miRNA
circRNA
mRNA
Spearman r
B
Top 43 unique ceRNA triplets by mean |r|
circRNA–mRNA
circRNA–miRNA
miRNA–mRNA
Spearman r
1.0
0.5
0.0
−0.5
−1.0
circRNA | miRNA | mRNA
Each circRNA, miRNA, and mRNA appears only once | Blue = negative | Red = positive | Sorted by mean |r|
C

Supplemental Figure S1 Legends. Regulatory landscape of circRNA and miRNA mediated gene expression. The regulatory landscape of circRNA-mediated gene expression, a comprehensive competing endogenous RNA (ceRNA) network was constructed based on brain circRNA–miRNA–mRNA interactions. 341 DEGs were identified as participating in significant ceRNA regulatory triads, each with more than 20 interaction connections, indicating high regulatory centrality. A) Heatmap showing the significant correlations of circRNA-miRNA and miRNA-mRNA. B) Heatmap illustrates the top unique ceRNA triplets by mean sum of r values for each interaction. For simplicity each of the circRNA, miRNA and mRNA appear only once. Blue indicates negative correlation; Red indicates positive correlation. C) Regulatory networks of circRNA-miRNA-mRNA in tau mice. The correlation network diagram visualizes the complex interaction between significant differentially enriched ncRNAs and mRNAs in cortical brain tissue of PS19 versus WT mice. The size of each node reflects its number of interactions in the network. Red lines indicate a positive correlation, where higher expressions of mRNA correspond to higher abundance circRNAs. Blue lines indicate a negative correlation, where higher expressions of miRNA correspond to lower expressions of mRNA or circRNAs. The darkness of the lines represents the strength of the correlation, light lines indicate lower correlation, and dark thick lines indicate higher correlation. The network includes the top 5% of interactions based on the sum of R for the three correlations. Blue boxes – miRNAs; green circles – circRNAs, and orange diamonds – mRNA.
